# The Influence of Mixing Speed on the Physicomechanical Parameters of Polyaddition Poly(dimethylsiloxanes) with Fillers

**DOI:** 10.3390/polym16172527

**Published:** 2024-09-06

**Authors:** Ewelina Chmielnicka, Małgorzata Szymiczek, Błażej Chmielnicki

**Affiliations:** 1 Łukasiewicz Research Network—Institute of Engineering of Polymer Materials and Dyes, M. Skłodowskiej-Curie St. 55, 87-100 Torun, Poland; blazej.chmielnicki@impib.lukasiewicz.gov.pl; 2 Institute of Theoretical and Applied Mechanics, Faculty of Mechanical Engineering, Silesian University of Technology, Konarskiego St. 18A, 44-100 Gliwice, Poland; malgorzata.szymiczek@polsl.pl

**Keywords:** polyaddition poly(dimethylsiloxane), thermography, surface resistivity

## Abstract

In this article, we present an analysis of the properties of polyaddition poly(dimethylsiloxanes) (PDMS) and their potential applications after modification. The focus is on understanding how different fillers and mixing speeds affect the mechanical and electrical properties of PDMS, as well as the benefits and challenges associated with these modifications. Additionally, the prospects for future development of PDMS-based technologies, which could bring significant innovations in various industrial fields, are discussed.

## 1. Introduction

Silicone polymers (silicones) constitute a group of polymer materials with exceptional physicochemical properties, making them widely used in technology and industry. Their unique properties result from their specific chemical structure. The basic structural element of silicones is a silicon-oxygen chain—[R_2_Si-O]-_n_, which forms the main polymer backbone. In this chain, silicon atoms are bonded with oxygen atoms, creating strong and stable bonds. Organic groups such as methyl (CH_3_), ethyl (C_2_H_5_), or phenyl (C_6_H_5_) are also attached to the silicon atoms [1,2,3]. The presence of these organic groups gives silicones specific properties that are difficult to achieve with other polymers. For example, methyl side groups increase the flexibility and thermal resistance of silicones [4], while phenyl groups can further improve their thermal stability and UV resistance [5]. Such a structure provides silicones not only with chemical stability but also with resistance to various external factors such as extreme temperatures, moisture, and chemicals.

One of the most important features of silicones is their resistance to extreme temperatures, allowing them to operate within a wide temperature range, from approximately −60 °C to +250 °C, making them ideal for the aerospace, automotive [6], and electronics industries [7,8]. Silicones with such properties include VMQ (Vinyl-Methyl-Polysiloxane) silicones, fluorosilicones (FVMQ), and phenyl silicones (PVMQ). Silicones such as HCR (High Consistency Rubber) are also chemically resistant and flexible across a wide temperature range, maintaining their mechanical properties [3]. Excellent insulating properties make them perfect for electrical and electronic applications as insulators and protective coatings and low gas permeability allows their use as gas barriers [9]. Silicones come in various forms, such as elastomers, gels, and resins, which have broad applications in industry and technology. LSR (Liquid Silicone Rubber) elastomers are flexible and durable, gels are used in medicine and electronics, and resins serve as protective coatings [10]. The low thermal and electrical conductivity of RTV (Room Temperature Vulcanizing) and HTV (High Temperature Vulcanizing) silicones are beneficial for insulation but can be a limitation when rapid heat dissipation or current conduction is required. In such cases, modifications increasing their conductivity are necessary.

Polyaddition poly(dimethylsiloxanes) (PDMS) are widely used polymers belonging to the group of organosilicon materials, sharing properties with materials of this type, while also standing out due to their low toxicity and biocompatibility [11]. These features make PDMS useful in many industrial fields, including medical applications such as contact lenses or implants [12]. They are also used in industries like food and cosmetics. One of the key advantages of PDMS is its versatility in chemical modifications. Adding various fillers can significantly alter its properties, enabling the use of PDMS in new, advanced technologies. An example is PDMS composites containing copper or graphite, which become electrical or thermal conductors. Such modifications open up new application possibilities, such as in the creation of flexible sensors, heated surfaces, or flexible electronics printing technology. The high thermal and chemical stability of PDMS makes it an ideal material for applications in extreme environmental conditions, further increasing its attractiveness in industrial applications.

Extensive research is currently being conducted on polydimethylsiloxane (PDMS) enriched with conductive fillers such as graphite and copper. The primary aim of this research is to modify the mechanical, electrical, and thermal properties of PDMS, thereby expanding its applications in advanced technologies.

One of the ongoing areas of research focuses on improving the electrical and thermal properties of PDMS. The addition of conductive fillers, such as copper or graphite, allows for the creation of composites that can be used as flexible electrical conductors or enhance PDMS’s ability to dissipate heat, which is crucial in electronics and heat dissipation systems. These materials are particularly useful in modern flexible electronics and sensors, where both flexibility and good electrical conductivity are required [13,14].

Due to the widespread use of silicones in industry, this issue is crucial for the practical application of this group of materials. Understanding the impact of the observed phenomena will allow for predicting the behavior of silicones modified with fillers and optimizing the technological processes of their production. Modifications of PDMS with conductive fillers such as graphite and copper contribute to the development of new applications and open up new possibilities for PDMS as an engineering material.

## 2. Materials and Methods

The studies were conducted on cast polyaddition poly(dimethylsiloxanes):

- RTV (Room Temperature Vulcanizing) 3428 (designation AA) from Elkem (Oslo, Norway), and
- RTV 3040SB (AB) (Elkem, Oslo, Norway), which can be used as molding materials for parts with complex geometry.

The selection of materials was made taking into account different viscosity values (RTV 3428 viscosity 20,000 mPa s and RTV 3040SB viscosity 35,000 mPa s), which are important for technological processes and applications. The properties of the materials are presented in Table 1.

Commercial materials were used. To minimize the impact of additives, they were not chemically modified in any way; only the tested fillers were introduced. The selected silicones belong to the group of polyaddition silicones, in which the crosslinking process results from the reaction of two base components, A and B. The combination of these components leads to the formation of a durable, crosslinked structure.

**Figure 1:**
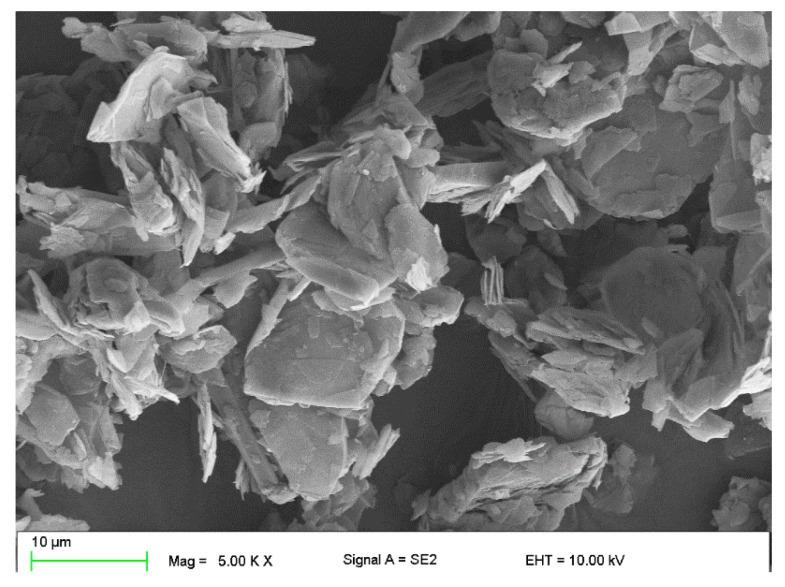
Microscopic image of graphite MG394.

**Figure 2:**
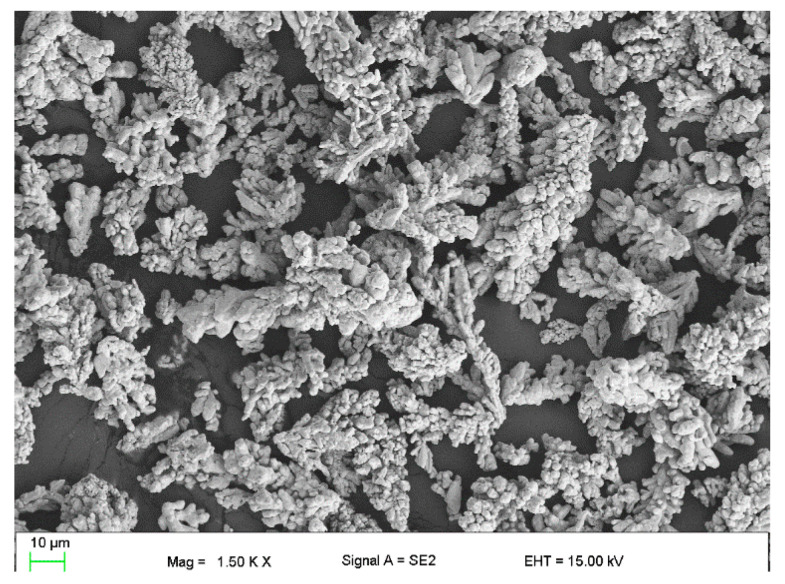
Microscopic image of copper LT16.

The modification of operational properties was carried out by a physical method through the introduction of the following fillers into the silicone matrix:

- Plate graphite MG 394 (with a particle size (flake) <45 μm) with thermal conductivity of 140–233 W/mK depending on the form—Figure 1.
- Copper powder (Lt16) with dendritic particle shape and particle size from 150 to 32 μm with thermal conductivity of 380 W/mK—Figure 2.

**Figure 3:**
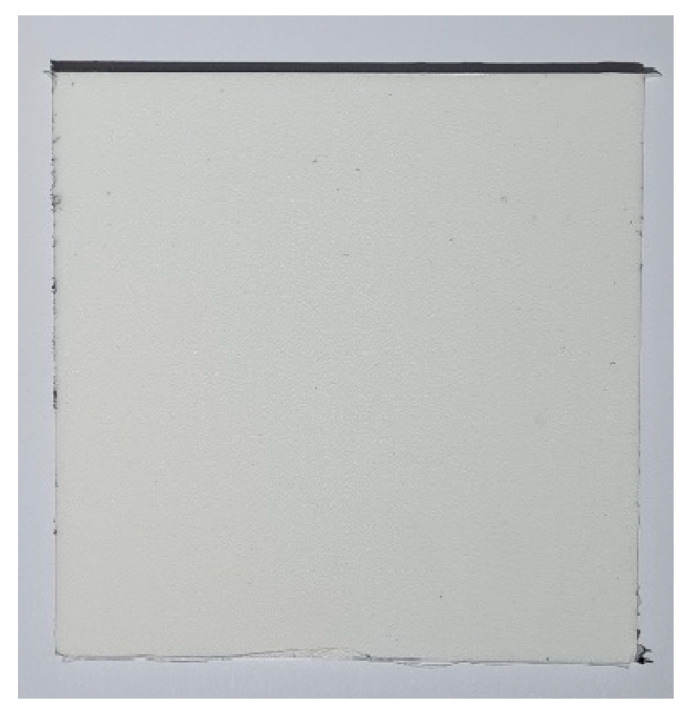
Sample PDMS AA 1500.

**Figure 4:**
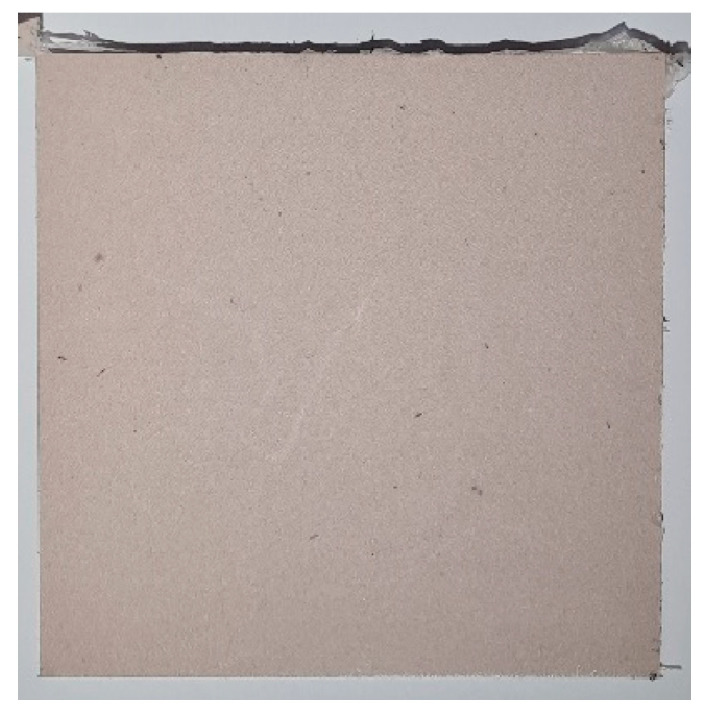
Sample PDMS AA 1500 2% Cu.

**Figure 5:**
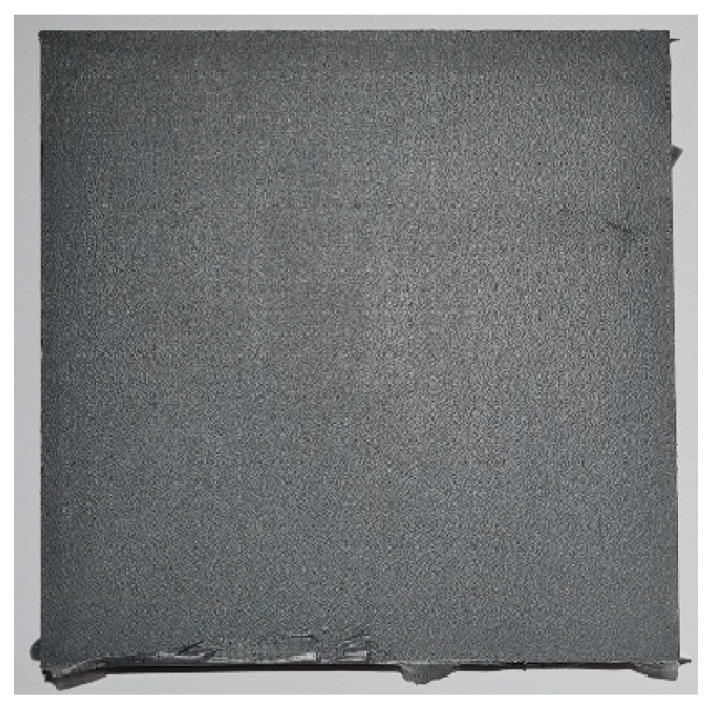
Sample PDMS AA 1500 4% graphite.

**Figure 6:**
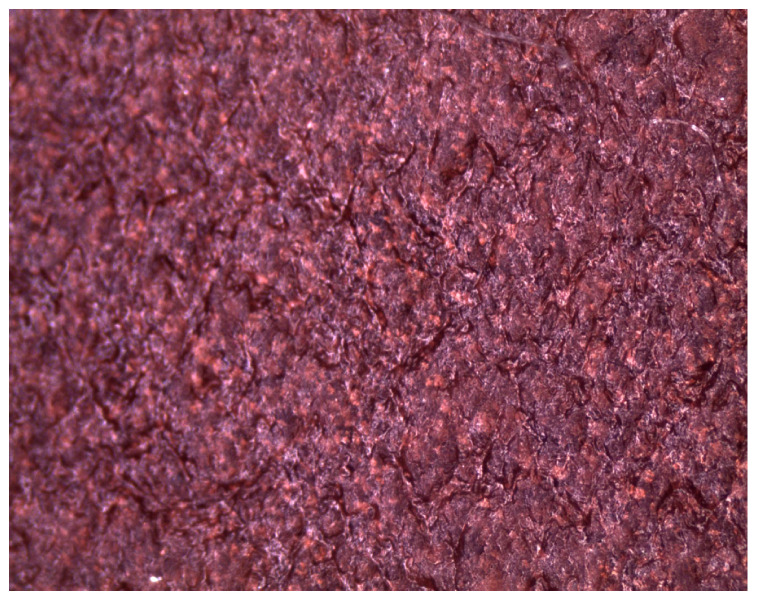
Cross-section of PDMS AB 1500 4% Cu.

**Figure 7:**
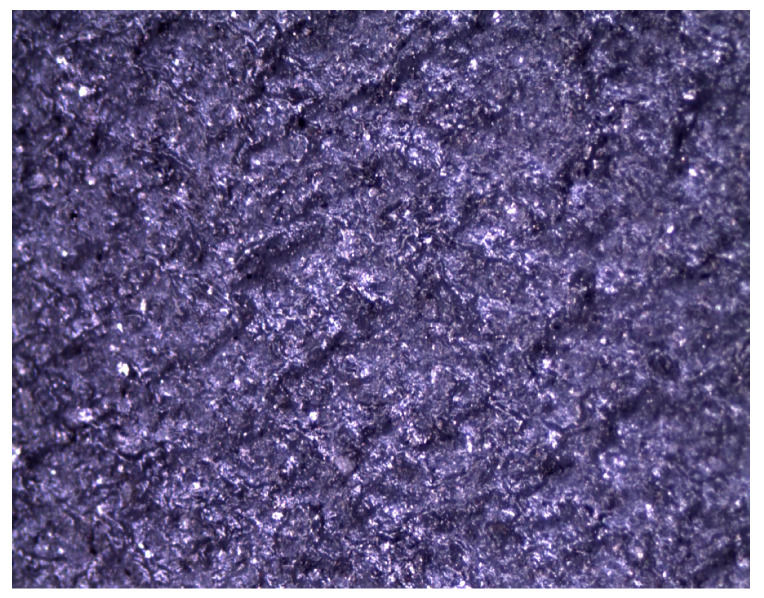
Cross-section of PDMS AB 1500 4% graphite.

The materials for the study were prepared under laboratory conditions, in accordance with the manufacturer’s recommendations regarding the content of the silicone base and catalyst (Table 1). The materials were mixed using a High-Speed Dissolver Dispermat LC30 mixer (VMA-Getzmann GMBH, Reichshof, Germany) at speeds of 500 and 1500 rpm. During the mixing process, the temperature of the produced PDMS and their composites was monitored. The intensive homogenization process led to significant aeration of the materials. To minimize the potential negative impact of discontinuities in their cross-section, the samples were degassed under a vacuum of 0.06 MPa for 5 min before casting, and then poured into polyethylene molds with dimensions of 100 × 100 × 4 mm. The prepared materials (Figure 3, Figure 4, Figure 5, Figure 6 and Figure 7) were conditioned at a temperature of 23 ± 5 °C for 24 h and then samples were cut according to the recommendations of the relevant standards.

Table 2 presents a summary of the names of the prepared samples according to their mixing speed and the fillers used.

### 2.1. Temperature Measurement

To perform thermal measurements during the mixing of silicones using the High-Speed Dissolver Dispermat LC30 mixer, a FLIR-E64501 thermal imaging camera connected with FLIR TOOLS software version 4.11.0 was used. This process aimed to monitor temperature changes during the mixing of silicones at two rotational speeds, 500 and 1500 rpm, and with selected fillers at two concentrations, 2% and 4%. The mixing process was conducted in polyethylene containers that were fixed in place to avoid external heating of the mixture, which could distort the accuracy of the results. Measurements using the thermal imaging camera were taken continuously during the mixing of the materials, with a frequency of 15 s. Temperature measurements were taken at a point halfway between the stirrer disc and the container wall, ensuring that the temperature of the mixed mass was measured, minimizing the influence of the stirrer and container walls on the result. To assess the effects of fillers and their concentrations on PDMS, the results were compared with samples without fillers (reference samples). The temperature measurement results were determined as the increase from each subsequent mixing time.

### 2.2. Density

The density of the materials was determined using the immersion method according to the PN-EN ISO 1183-1:2019-05 standard [16] using a Mettler-Toledo XS105 DualRange analytical balance with a density measurement attachment (Mettler-Toledo GmbH, Schwerzenbach, Switzerland). The study was conducted in distilled water at a temperature of 25 ± 0.5 °C. Tests were carried out on 9 samples measuring 10 × 10 mm, taken from different areas of the cast plates.

### 2.3. Hardness

The hardness of the materials was determined using the Shore A method according to the PN-EN ISO 868:2005 standard [17] using a Zwick&Roell Shore A durometer (Zwick GmbH & Co., Ltd., Ulm, Germany). Ten hardness measurements were taken on each sample with dimensions of 100 × 100 mm.

### 2.4. Mechanical Testing

Tensile strength tests were conducted on five type 5A samples according to the methodology of the PN-EN ISO 527-2 standard [18]. The samples were prepared by cutting them with a cutter to ensure structural continuity (no notches), eliminating the influence of potential notches. Tests were conducted on a LaborTech LabTest 6.100 (LaborTech, Opava, Czech Republic) tensile testing machine equipped with mechanical grips to ensure stable sample holding. Tested features, such as tensile stress at break and elongation at break, were determined using a LaborTech ONE2 video extensometer (LaborTech, Opava, Czech Republic) according to the PN-EN ISO 527-1 [19] and PN-EN ISO 527-2 standards. The tensile speed was 100 mm/min. Tests were conducted in a climate-controlled room at an ambient temperature of 25 ± 2 °C and humidity of 50 ± 2%.

### 2.5. Surface Resistivity

The surface resistivity was measured on a sample with dimensions of 100 mm × 100 mm × 4 mm. The samples were placed under laboratory conditions for 24 h and their surfaces were cleaned with acetone before testing. The tests were conducted using a Keithley model 8009 resistivity chamber connected to a Keithley 6517A electrometer (Keithley, Bracknell, UK). Measurements were performed at a voltage of 100 V.

## 3. Results

### 3.1. Temperature Measurement

**Figure 8:**
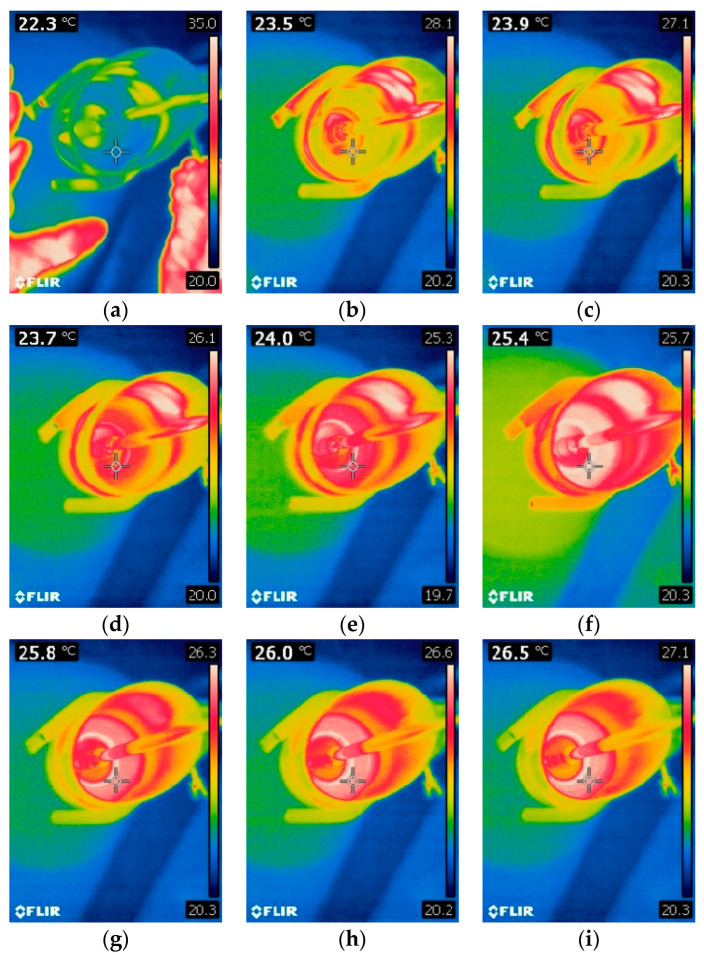
Temperature changes during mixing for AB 500. (**a**) Start of mixing, (**b**) Time 15 s, (**c**) Time 30 s, (**d**) Time 45 s, (**e**) Time 60 s, (**f**) Time 75 s, (**g**) Time 90 s, (**h**) Time 105 s, (**i**) Time 120 s.

**Figure 9:**
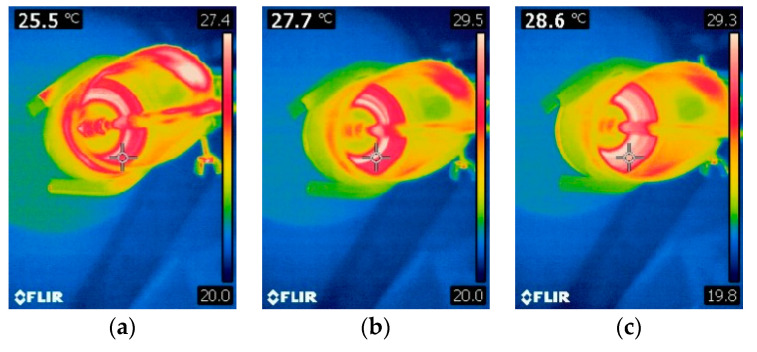

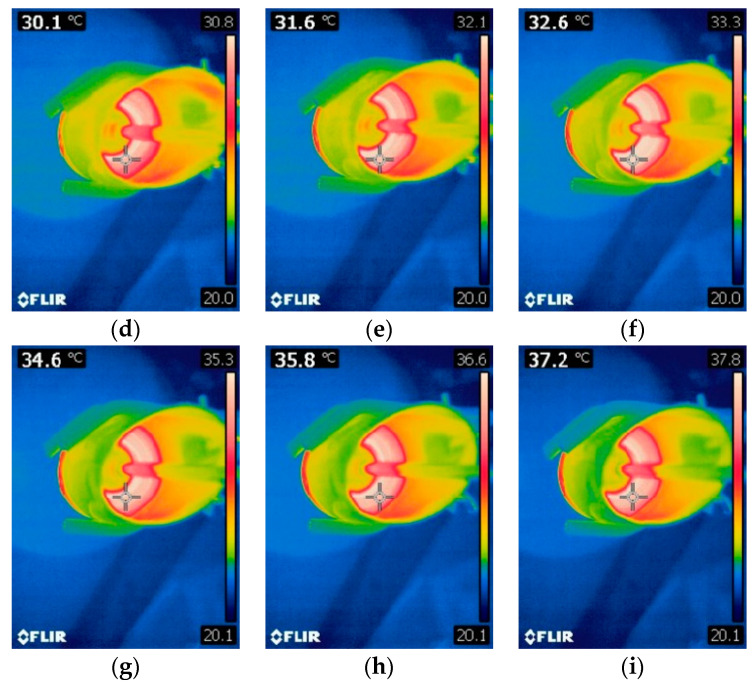
Temperature changes during mixing for AB 1500. (**a**) Start of mixing, (**b**) Time 15 s, (**c**) Time 30 s, (**d**) Time 45 s, (**e**) Time 60 s, (**f**) Time 75 s, (**g**) Time 90 s, (**h**) Time 105 s, (**i**) Time 120 s.

Figure 8a–i and Figure 9a–i present thermal images of temperature measurements during the mixing process of commercial PDMS AB 500 and AB 1500 materials without fillers, used according to the supplier’s recommendations (mixing speed 500 and 1500 rpm, respectively). The figures show images taken every 15 s from the start of mixing until its completion. The measurements are presented for mixing speeds of 500 and 1500 rpm for AB silicone.

Based on the conducted measurements, it can be concluded that the mixing speed affects the temperature increase of the mixed materials. At a speed of 1500 rpm, the temperature increase is higher, which may accelerate chemical changes in the material, leading to a shorter lifespan of the materials and limiting their moldability. Temperature measurements are a crucial part of the study due to the reduced working time for casting PDMS into molds. This also affects the feasible mixing time required to achieve a homogeneous material throughout the sample cross-section. Proper homogenization, especially for samples with conductive fillers such as graphite or copper, directly impacts the properties obtained after forming and curing the samples. The formation of efficient percolation paths and the reduction of surface resistivity depend on this process. From the perspective of mechanical testing, the homogenization process is important for the precise distribution of the filler in the matrix, preventing the formation of agglomerates that could become weak points during strength testing and negatively affect the material’s strength.

**Figure 10:**
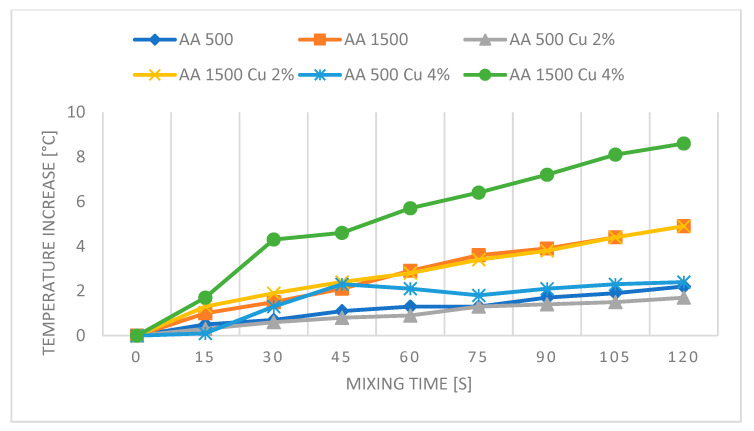
Graph for PDMS AA—reference samples and with Cu.

**Figure 11:**
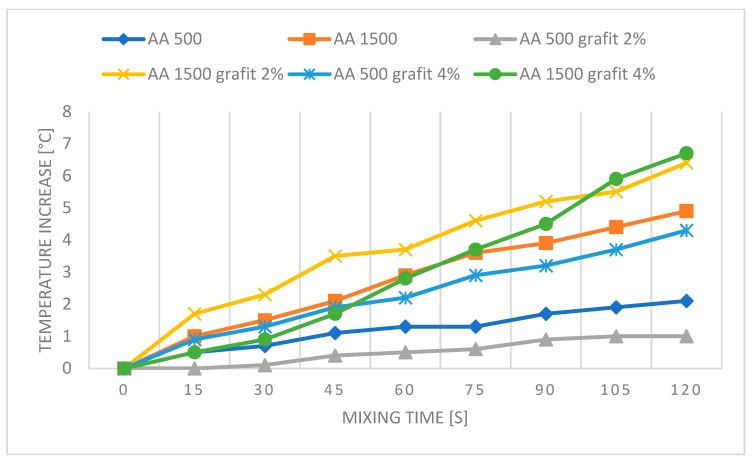
Graph for PDMS AA—reference samples and with graphite.

**Figure 12:**
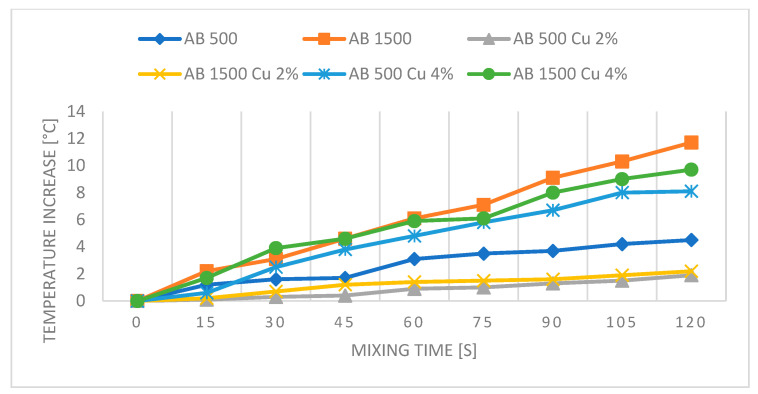
Graph for PDMS AB—reference samples and with Cu.

**Figure 13:**
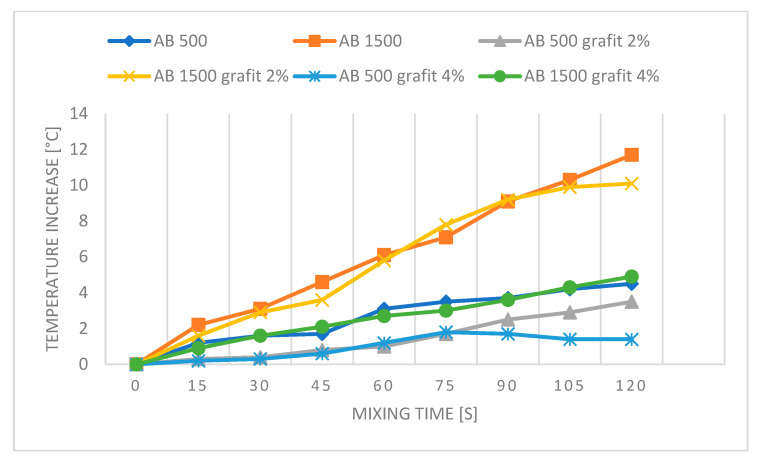
Graph for PDMS AB—reference samples and with graphite.

Figure 10, Figure 11, Figure 12 and Figure 13 present graphs showing the influence of mixing speed on samples based on AA and AB silicones at speeds of 500 and 1500 rpm with Cu and graphite fillers at 2% and 4% concentrations. The graphs are based on thermal imaging data and show the temperature increase relative to the mixing time.

The presented results show the influence of copper powder and graphite additives on the temperature increase of AA and AB silicone mixtures subjected to mixing at speeds of 500 and 1500 rpm. The differences in temperature increase are due to both the type of silicone used and the concentration of the added fillers.

For AA silicones, the pure AA 500 mixture at a speed of 500 rpm shows a moderate temperature increase to 2.2 °C, while at a speed of 1500 rpm, AA 1500 reaches a temperature of 4.9 °C. The reduction in temperature increase for the AA 500 Cu 2% sample and the comparable temperature increase of the AA 1500 Cu 2% sample may suggest the effectiveness of the filler in dissipating heat, indicating a slight decrease or stabilization in temperature. Slight temperature increases for samples with 4% copper content likely indicate increased friction within the material.

For AB silicones, the pure AB 500 mixture at a speed of 500 rpm shows a significant temperature increase to 4.5 °C after 2 min, while increasing the mixing speed to 1500 rpm results in a temperature of 11.7 °C, likely due to the higher viscosity of the silicone and the generation of greater friction. The temperature fluctuations of samples with copper filler are similar to those of AA silicone samples.

For AA silicones, the reference AA 500 sample shows a moderate temperature increase to 2.2 °C. Adding 2% graphite reduces the temperature increase to 1 °C, which may be due to the lubricating and thermal conductivity properties of graphite. The addition of 4% graphite causes a temperature increase to 4.3 °C, which could be related to the viscosity of the matrix in which it is dispersed. Graphite particles, due to their lubricating properties, likely form agglomerates at low speeds. The centrifugal force causes a situation in a complex measurement system where a middle layer with a higher graphite content may form, leading to an increase in temperature compared to the reference sample. The higher filler content likely also contributes to increased friction between the filler itself and the mixer.

At a speed of 1500 rpm, the pure AA 1500 reaches a temperature increase of 4.9 °C. Adding 2% graphite results in a significant temperature increase to 6.4 °C, which may be due to the limited effectiveness of graphite in dissipating heat at higher speeds. Adding 4% graphite causes a temperature increase to 8.7 °C, indicating increased friction and heat generation at higher concentrations.

For AB silicones, the pure AB 500 mixture at a speed of 500 rpm shows a significant temperature increase to 4.5 °C, likely due to the higher viscosity of the material compared to AA silicone, which showed half the temperature increase for the reference sample at the same speed. Adding 2% graphite reduces the temperature increase by 3.5 °C compared to the reference sample. This change may be due to the lubricating properties of graphite. Adding 4% graphite further reduces the temperature increase to only 1.4 °C, likely due to both the lubricating and conductive properties of the graphite filler.

At a speed of 1500 rpm, the reference AB 1500 sample reaches a temperature increase of 11.7 °C. Adding 2% graphite causes a significant temperature increase of 10.1 °C, suggesting that graphite is not effective in dissipating heat at higher speeds in a higher viscosity matrix compared to the AA sample. The higher viscosity may contribute to increased friction between the mixture and the mixer. Adding 4% graphite reduces the temperature increase by 4.9 °C, indicating that a higher concentration of graphite can better manage heat dissipation even at higher mixing speeds. The lubricating properties of graphite are also likely more noticeable with a higher filler content and higher mixing speed.

### 3.2. Density

**Figure 14:**
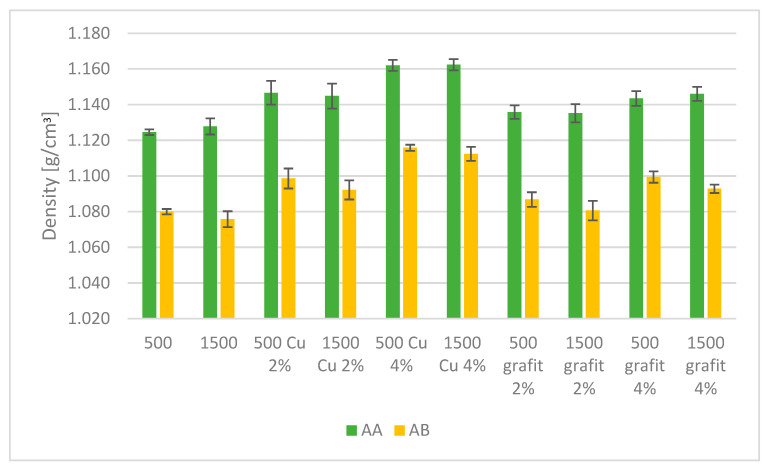
Density measurement of polyaddition PDMS AA and AB.

Figure 14 presents the average density values obtained for samples with various fillers and mixing speeds. The presented results are the average values from 10 measurements, with error bars indicating the standard deviation of the obtained values.

Based on the obtained research results (Figure 14), lower density values were observed for PDMS AB compared to the reference sample PDMS AA. Regarding the mixing speed, the changes in density values are comparable and fall within the measurement error margin. The content of the filler had the greatest impact on the obtained results. The highest density values were recorded for samples with a 4% filler content compared to the reference samples for both PDMS types, which is likely related to the specific gravity of the filler.

### 3.3. Hardness

**Figure 15:**
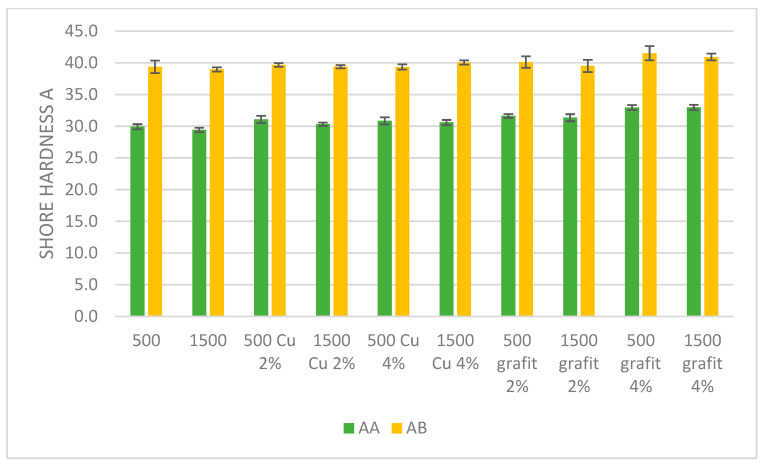
Shore hardness A of polyaddition PDMS AA and AB.

Figure 15 presents the average Shore A hardness values obtained for samples with various fillers and mixing speeds. The presented results are the average values from 10 measurements, with error bars indicating the standard deviation of the obtained values.

The conducted studies aimed to assess the influence of copper powder and graphite additives on the Shore A hardness of AA and AB silicone mixtures mixed at speeds of 500 and 1500 rpm. The results indicate differences in hardness depending on the type and concentration of fillers as well as the mixing speed.

The addition of 2% copper to AA 500 increases the average hardness to 31.1 °Sh A, and for AA 1500 to 30.3 °Sh A. In contrast, the addition of 4% copper to AA 500 raises the hardness to 30.9 °Sh A, while for AA 1500 the hardness is 30.6 °Sh A. The increase in hardness in the presence of copper suggests that this additive affects the crosslinking and mechanical properties of the silicone, although the effects are less pronounced than in the case of AB silicones.

The AA 500 sample with 2% graphite increases the average hardness to 31.6 °Sh A, and for AA 1500 to 31.4 °Sh A. On the other hand, the addition of 4% graphite significantly increases the hardness of AA 500 to 32.9 °Sh A, and for AA 1500 to 33.0 °Sh A. The increase in hardness is more pronounced in the case of graphite, which can be attributed to its mechanical reinforcement properties, similar to AB silicones.

The addition of 2% copper to the AB 500 mixture slightly increases the average hardness to 39.7 °Sh A, while for AB 1500 the average hardness is 39.4 °Sh A. Similarly, the addition of 4% copper to AB 500 slightly decreases the hardness to 39.3 °Sh A, while for AB 1500 the hardness increases to 40.1 °Sh A. These changes suggest that copper influences the crosslinking structure of the silicone, increasing its hardness, especially at higher concentrations and mixing speeds.

The AB 500 sample with 2% graphite increases the average hardness to 40.1 °Sh A, while for AB 1500 it is 39.5 °Sh A. On the other hand, the addition of 4% graphite significantly increases the hardness of AB 500 to 41.5 °Sh A, and for AB 1500 to 40.9 °Sh A. Higher concentrations of graphite increase hardness, which may be due to the mechanical reinforcement properties of this filler.

### 3.4. Mechanical Testing

**Figure 16:**
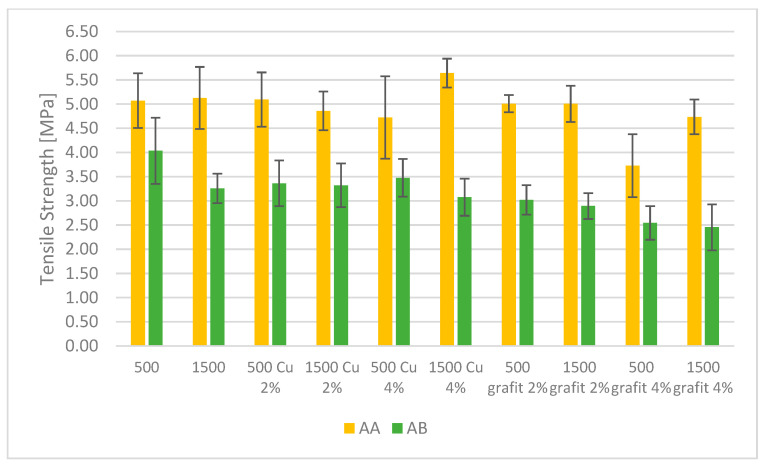
Tensile Strength of polyaddition PDMS AA and AB.

**Figure 17:**
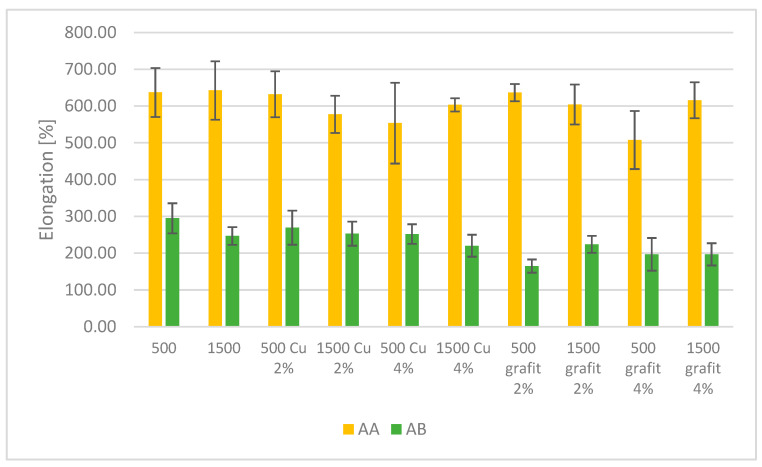
Relative Elongation of polyaddition PDMS AA and AB.

Figure 16 and Figure 17 present the average tensile strength values and relative elongation obtained for samples with various fillers and mixing speeds. The presented results are the average values from 5 measurements, with error bars indicating the standard deviation of the obtained values.

Based on the results, it can be observed that PDMS AA exhibits higher tensile strength compared to the AB series. For the reference samples of PDMS AA, no effect of mixing speed on the tensile strength values was observed. The impact of mixing speed can be seen for the reference sample of PDMS AB, where there is a slight decrease in tensile strength for the AB 1500 sample compared to AB 500. The highest tensile strength among the tested materials is exhibited by AA 1500 Cu 4%, achieving an average value of 5.64 MPa. This suggests that the addition of 4% dendritic copper significantly improves the mechanical strength of this material, which may be related to the level of filler dispersion in the matrix and its surface area, which is developed and promotes mechanical bonding between the poly(dimethylsiloxane) matrix and the filler. In the case of a developed filler surface with appropriately low polymer viscosity, a phenomenon occurs where the filler particles mechanically bond with the material by infiltrating irregularities and forming an additional connection between the filler and the polymer.

The filler in the form of graphite (both 2% and 4%) reduces the tensile strength in both types of PDMS. In the case of PDMS AB, AB 500 graphite 4% achieves the lowest tensile strength value of 2.55 MPa.

In terms of relative elongation, PDMS AA achieves the highest values. Silicones with Cu additives also exhibit high elongation, particularly AA 500 Cu 2%, which reaches an average value of 632.2%. Similar to tensile strength, graphite additives reduce relative elongation. PDMS AB 500 graphite 2% achieves an average value of 164.9%, which is significantly lower compared to other tested materials. Samples based on PDMS AB show lower relative elongation values compared to PDMS AA, indicating the lower flexibility of these materials.

Based on the conducted studies, different behaviors of the two tested types of PDMS can be observed. In the case of PDMS AA, which initially has greater flexibility, no significant decrease in tensile strength was observed for mixtures containing fillers. In contrast, the introduction of fillers into PDMS AB resulted in a decrease in tensile strength values. This effect is most likely related to the lower flexibility and higher viscosity of the AB sample. Due to its lower flexibility, this material is more prone to damage induced by the presence of filler particles compared to the more flexible AA material.

### 3.5. Surface Resistivity

**Figure 18:**
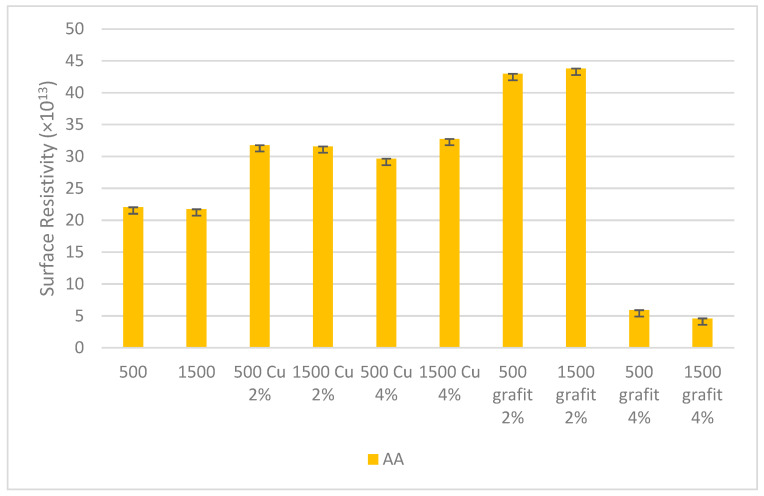
Surface resistivity of polyaddition PDMS AA.

**Figure 19:**
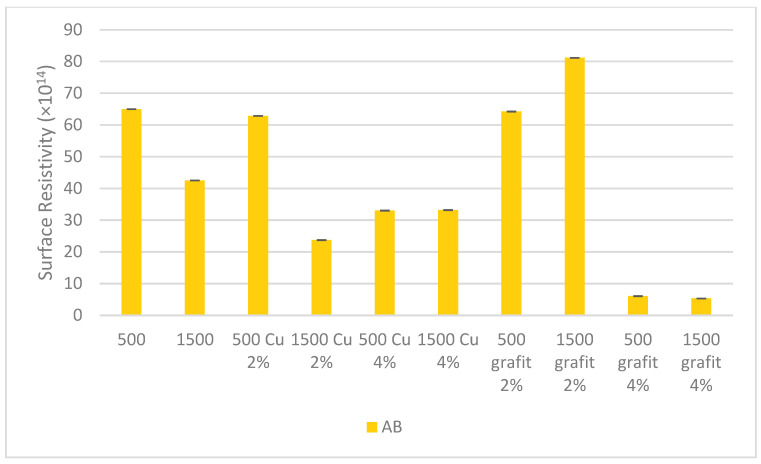
Surface resistivity of polyaddition PDMS AB.

Figure 18 and Figure 19 present the surface resistivity values obtained for samples with various fillers and mixing speeds.

Based on the obtained surface resistivity measurement results, it was observed that the mixing speed did not affect the results for the tested samples. Changes were observed for samples with higher filler content in the silicone matrix. Significant changes are visible for samples AA 500 Cu 4%, AA 1500 Cu 4%, AB 500 graphite 4%, and AB 1500 graphite 4%, where the surface resistivity value decreased by an order of magnitude. The reduction in surface resistivity is significant for achieving electrical or thermal conductivity in polyaddition poly(dimethylsiloxane). The obtained results provide a basis for further research aimed at creating efficient percolation paths, and consequently, obtaining a conductive organosilicon material.

## 4. Conclusions

The obtained density measurement results indicate the influence of the amount and type of filler on the density value. The highest density increase was observed with the use of 4% Cu filler, which is likely related to its specific gravity. The change in mixing speed did not have a noticeable effect on the density value of the crosslinked material. The density differences for individual samples with the same filler content fall within the margin of error of the testing method.

The analysis of the results shows that both copper powder and graphite additives affect the hardness of AA and AB silicone mixtures. Copper, as a filler, generally increases hardness, especially at higher concentrations and mixing speeds. The impact of graphite is even more pronounced, suggesting that graphite acts as an effective reinforcing agent, increasing the hardness of the mixture. Higher mixing speed (1500 rpm) generally leads to greater changes in hardness, which may result from a more intensive mixing process and better dispersion of fillers in the silicone matrix, or the impact of temperature on more effective crosslinking of the silicone matrix.

Based on the presented data, it can be concluded that AA series silicones, especially those with Cu additives, exhibit the best mechanical properties in terms of both tensile strength and relative elongation. Graphite additives have a negative impact on these properties, reducing both the strength and flexibility of the materials. AB series silicones have lower strength and flexibility compared to the AA series, which may limit their use in demanding applications. The change in mixing speed did not have a noticeable effect on the mechanical properties of the crosslinked material. The differences in tensile strength and relative elongation for individual samples with the same filler content fall within the margin of error of the testing method.

For surface resistivity measurements, a decrease in this value was observed for materials with the highest filler content. The results indicate that a higher amount of filler allows for the creation of conductive paths in the silicone matrix, which is a hint for further research in this area to achieve more satisfactory results.

The analysis shows that both copper powder and graphite additives affect the dynamic temperature increase of silicone mixtures. Copper, as a good heat conductor, generally reduces the temperature increase, but higher concentrations may lead to increased friction and heat generation. Graphite, due to its lubricating properties, reduces the temperature increase, but its effectiveness may be limited at higher mixing speeds and higher concentrations. The mixing speed significantly affects the temperature increase, and the appropriate selection and concentration of fillers are crucial for optimizing the silicone crosslinking process and ensuring the desired properties of the final product.

Based on the conducted studies, it can be concluded that the mixing speed affects the temperature increase of the mixture. At 1500 rpm, a significantly higher temperature increase can be observed compared to homogenization at 500 rpm. The temperature differences are most noticeable in the reference samples, where the influence of the filler on the polymer matrix and the processes occurring during mixing are excluded.

Viscosity of PDMS influenced the temperature increase in the samples and the behavior of the introduced filler. Higher viscosity generated greater friction between the stirrer and the mixture. Additionally, viscosity affected the distribution of the filler in the matrix and the formation of agglomerates or better homogenization of the mixture, resulting in the filler likely dissipating heat more effectively.

## Figures and Tables

**Table 1:** Material properties based on manufacturer data [15].

Properties	Standards	Unit	AA	AB
Mixing proportion (base:catalyst)	-	-	100:10	100:10
Density 25 °C	ISO 1183	g/cm^3^	1.12	1.08
Brookfield viscosity	-	mPa s	20,000	35,000
Life time 25 °C	-	min	60	75
Demoulding time 23 °C	-	h	16	24

Dependent on the catalyst.

**Table 2:** Designation of samples subjected to testing.

Designation	Mixing Speed [rpm]	Type of Filler	Amount of Filler [%]
AA 500	500	none	none
AA 1500	1500	none	none
AA 500 Cu 2%	500	copper powder	2
AA 1500 Cu 2%	1500	copper powder	2
AA 500 Cu 4%	500	copper powder	4
AA 1500 Cu 4%	1500	copper powder	4
AA 500 graphite 2%	500	plate graphite	2
AA 1500 graphite 2%	1500	plate graphite	2
AA 500 graphite 4%	500	plate graphite	4
AA 1500 graphite 4%	1500	plate graphite	4
AB 500	500	none	none
AB 1500	1500	none	none
AB 500 Cu 2%	500	copper powder	2
AB 1500 Cu 2%	1500	copper powder	2
AB 500 Cu 4%	500	copper powder	4
AB 1500 Cu 4%	1500	copper powder	4
AB 500 graphite 2%	500	plate graphite	2
AB 1500 graphite 2%	1500	plate graphite	2
AB 500 graphite 4%	500	plate graphite	4
AB 1500 graphite 4%	1500	plate graphite	4

## Data Availability

The original contributions presented in the study are included in the article, further inquiries can be directed to the corresponding author.
